# Prevalence of Chronic Kidney Disease and Associated Factors among the Diabetic and Prediabetic Population in the Bandare-Kong Cohort Study; A Population-Based Study

**DOI:** 10.34172/aim.31194

**Published:** 2024-09-01

**Authors:** Masoumeh Kheirandish, Ebrahim Eftekhar, Abnoos Azarbad, Elaheh Salarpour, Mehdi Shahmoradi, Sara Ghazizadeh, Alireza Malektojari, Zohre Nikeghbali, Soheil karimi Lengeh, Aghdas Dehghani

**Affiliations:** ^1^Endocrinology and Metabolism Research Center, Hormozgan University of Medical Sciences, Bandar Abbas, Iran; ^2^Molecular Medicine Research Center, Hormozgan Health Institute, Hormozgan University of Medical Sciences, Bandar Abbas, Iran; ^3^Evidence Based Medicine Center, Hormozgan University of Medical Sciences, Bandar Abbas, Iran; ^4^Cardiovascular Research Center, Hormozgan University of Medical Sciences, Bandar Abbas, Iran; ^5^Student Research Committee, Hormozgan University of Medical Sciences, Bandar Abbas, Iran

**Keywords:** Chronic kidney diseases, Diabetes mellitus, Hypertension, Prediabetes

## Abstract

**Background:**

This investigation aims to examine the relationship between diabetes and prediabetes with chronic kidney disease (CKD) while taking into account key risk factors such as gender, age, lifestyle, smoking habits, and blood pressure.

**Methods:**

Between November 17, 2016, and November 22, 2018, 4063 subjects aged 35 to 70 years were enrolled in the first phase of the Bandare-Kong Non-Communicable Disease (BKNCD) Cohort Study, which is part of the PERSIAN (Prospective Epidemiological Research Studies in IrAN) cohort and was conducted in a coastal region of the Hormozgan province in southern Iran. CKD was calculated using the Modification of Diet in Renal Disease (MDRD) formula based on glomerular filtration rate (GFR)<60 mL/min per 1.73 m^2^, or albumin/Cr>30 mg/g in random urine, self-reported kidney failure, or dialysis. Urine albumin and creatinine were determined by standard kits (Pars Azmoon, Tehran, Iran) and the BT1500 automatic chemistry analyzer (Biotecnica Instruments, Rome, Italy).

**Results:**

The prevalence of CKD was found to be 15.3%, with 29.6% identified in diabetic individuals and 16.5% in prediabetic patients. So, the prevalence of CKD in diabetics was higher than prediabetics and normal people. Increased age, dysglycemia (diabetes or prediabetes), hypertension, and use of angiotensin receptor blockers were markedly associated with an elevated risk of CKD in adults.

**Conclusion:**

The study emphasizes the importance of early detection and management of CKD risk factors, particularly among high-risk individuals, to mitigate CKD progression and associated complications. By addressing modifiable risk factors, proactive screening, and enhanced awareness, significant strides can be made in reducing CKD burden and improving patient outcomes.

## Introduction

 Chronic kidney disease (CKD) is characterized by a persistent reduction in kidney function, the presence of markers indicating kidney damage, or both, lasting for a minimum of three months, regardless of the underlying cause.^1^ The rising number of risk factors and other non-communicable diseases contributes to an alarming increase in the population affected by CKD, posing a significant challenge globally.^2^ Estimates suggest that CKD prevalence ranges between 11% and 13% worldwide, with the majority in low- to middle-income nations.^3^ Approximately 752.7 million individuals globally are affected by CKD, with 5 to 10 million deaths attributed annually to kidney disease.^4,5^ Projections indicate a rise in CKD-related mortality, with an anticipated global rank of 5th in terms of years of life lost (YLL) by 2040, up from 16th in 2016.^6^

 There is a strong correlation between CKD and increased risk of death and cardiovascular events.^7^ While the exact cause of CKD remains elusive, researchers have found various risk factors to contribute to its development, including male gender, obesity, aging, hypertension, diabetes mellitus, hyperlipidemia, a family history of renal disease, smoking, excessive alcohol drinking, HIV infection, electrolyte disorders, low-income occupations, physical inactivity, urbanization, and population growth.^8-14^

 Diabetes and hypertension collectively account for over 60% of end-stage kidney disease (ESKD) cases globally, making them the predominant causes of CKD worldwide.^3^ Specifically, diabetes contributes to 30% to 50% of all CKD and ESKD cases worldwide, significantly increasing the risk of cardiovascular complications, mortality, kidney failure, and escalating healthcare expenses.^15,16^ Consequently, early screening based on estimated glomerular filtration rate (eGFR) is recommended for diabetic patients to facilitate prompt identification and treatment of CKD.^17^

 However, data scarcity persists in the Middle East regarding CKD prevalence among diabetic individuals, and there is considerable variability across other available studies. For example, the prevalence of CKD among diabetic patients is reported to be 38.5% in Palestine,^18^ 34.7% in Morocco,^19^ 24.6% in South Africa,^20^ and 18.2% in Ethiopia.^21^ Research indicates that early identification and management of diabetes, hypertension, and other chronic conditions can enhance renal outcomes and may decrease or even prevent the progression of chronic CKD.^22^

 Although diabetes is a well-established risk factor for CKD, uncertainty surrounds whether prediabetes also contributes to kidney function impairment and CKD development. Research from the Framingham Heart Study offspring cohort has suggested that cardiovascular risk factors like blood pressure, plasma lipid levels, gender and age may be stronger predictors of CKD than prediabetes itself.^23^ In contrast, findings from a cross-sectional study in the Cooperative Health Research in the Augsburg Region (KORA) highlighted the potential negative impact of prediabetes on kidney health.^24^ Additionally, studies on the Chinese population have reported conflicting evidence regarding the link between prediabetes and CKD.^25,26^ The relationship between prediabetic glycemic indices and CKD remains uncertain, with different studies yielding inconsistent results.^24,27-30^

 Given that lifestyle modifications and pharmacological interventions can prevent or mitigate diabetes and its complications in high-risk subjects, understanding the potential association between prediabetes and CKD becomes crucial.^31^ Identifying reliable glycemic measurements predictive of increased CKD risk in prediabetic individuals becomes imperative, considering the escalating global prevalence of CKD and its high mortality rate. Such insights could pave the way for novel CKD treatments and more robust preventive measures for individuals with prediabetes and diabetes. The aims of this investigation were to explore the relationship between diabetes, prediabetes, and CKD, considering essential risk factors, including gender, age, lifestyle, smoking habits, and blood pressure.

## Materials and Methods

 This study represents the initial phase of the Bandare-Kong Non-Communicable Disease (BKNCD) Cohort Study, conducted in a coastal region of the Hormozgan province, southern Iran, as part of the larger PERSIAN (Prospective Epidemiological Research Studies in IrAN) cohort. The methodology has been previously published.^32^ Between November 17, 2016, and November 22, 2018, a total of 4063 participants aged 35 to 70 years were enrolled. After excluding incomplete records and pregnant women, the final analysis included 3642 participants. Trained personnel collected sociodemographic data, such as marital status, age, gender, employment status, education level, smoking habits, wealth score index (WSI), and physical activity, through face-to-face interviews. Body weight was determined using a mechanical scale, with participants wearing minimal clothing and no shoes, and accuracy to 0.5 kg. Height was measured with participants standing barefoot, in a natural shoulder position. Waist circumference (WC) was assessed at the midpoint between the iliac crest and the last palpable rib, with each measurement taken twice per participant, and the average value was noted. All measurements were performed using a stretch-resistant tape, with accuracy recorded to the nearest 0.5 cm.

 Cigarette smoking status was determined using self-reported data. “Current smokers” were defined as individuals who were actively smoking or had smoked 100 or more cigarettes in their lifetime. “Ex-smokers” were those who had also smoked at least 100 cigarettes but had not smoked within the last six months. Physical activity was assessed by recording 24-hour activity levels, encompassing work, exercise, and leisure activities. These were calculated on a weekly basis and categorized into low, moderate, and vigorous intensity levels. The wealth status index (WSI) was calculated using multiple correspondence analysis, incorporating variables such as ownership of appliances, travel history, and assets. The WSI was then divided into quintiles ranging from “very rich” to “very poor.” Quantitative data were expressed as means and standard deviations, while qualitative data were reported as proportions and frequencies.

 CKD was calculated using the Modification of Diet in Renal Disease (MDRD) formula based on GFR < 60 mL/min per 1.73 m^2^, or albumin/Cr > 30 mg/g in random urine, self-reported kidney failure, or dialysis.

 Urine albumin and creatinine were measured by standard kits (Pars Azmoon, Tehran, Iran) and the BT1500 automatic chemistry analyzer (Biotecnica Instruments, Rome, Italy).

 Normal distribution of the data was checked using Kolmogorov-Smirnov test and Q-Q graph tests. In both cases, the normality of the data distribution was confirmed. For calculation of the descriptive statistics of CKD prevalence, independent *t* test, and Mann-Whitney test were used for normally and non-normally distributed quantitative data, respectively. Chi-square tests, independent *t*-tests, and one-way analysis of variance tests, compared quantitative and qualitative variables between groups; the assumption of independent *t* tests and one-way analysis of variance tests is normality and homogeneity of variance that have been checked. Logistic regression examined the relationship between chronic renal failure, diabetes, prediabetes, and other risk factors using crude and multivariate models, although the assumption of linearity of the logistic regression model was evaluated for quantitative predictors. Variables with a *P* value of ≤ 0.2 in the univariate analysis were included in the multivariate logistic regression model, employing the “Wald” method. A *P* value of < 0.05 was considered as statistically significant. SPSS version 22.0 was used for analysis.

## Results

 A total of 3642 individuals participated in this study. Of the total survey subjects, 1610 were men (44.2%), and 2032 were women (55.8%). The average age of people with CKD (50.67 ± 9.84 year) was higher than that of people without CKD (47.67 ± 9.05 year). The waist circumference of people with/without CKD was almost equal. The prevalence of CKD in diabetics (29.6%) was higher than prediabetics (16.5%) and normal (10.5%) people (Tables 1, 2).

**Table 1:** Frequency Distribution of Demographic Variables of the Study Subjects with CKD Status

**Variables**	**Normal**		**Prediabetes**		**Diabetes**	
	**CKD**		**CKD**		**CKD**	
	**No**	**Yes**	**No**	**Yes**	**No**	**Yes**
Age (y) (Mean ± SD)	45.78 ± 8.57	52.29 ± 10.25	48.21 ± 8.75	53.51 ± 9.46	52.81 ± 8.70	56.09 ± 8.02
*P *value	< 0.001		< 0.001		< 0.001	
Waist Circumference (cm)	91.56 ± 11.93	93.99 ± 12.24	95.94 ± 11.40	96.17 ± 11.22	97.41 ± 11.20	97.62 ± 10.43
*P *value	0.003		0.83		0.82	
GFR (ml/min)	81.07 ± 11.74	63.32 ± 15.46	80.39 ± 12.19	60.63 ± 12.68	78.86 ± 11.50	65.80 ± 18.07
*P *value	< 0.001		< 0.001		< 0.001	
Urine Alb/Cr ratio (mg/g)	2.33 ± 0.12	44.48 ± 6.19	2.34 ± 0.20	44.69 ± 7.67	4.83 ± 0.34	146.91 ± 17.34
*P *value	< 0.001		< 0.001		< 0.001	
Gender, N (%)						
Female	1089 (89.9)	123 (10.1)	331 (84.7)	60 (15.3)	294 (68.5)	135 (31.5)
Male	875 (89.0)	108 (11.0)	307 (82.3)	66 (17.7)	187 (73.6)	67 (26.4)
*P *value	0.53		0.38		0.16	
Hypertension (%)						
No	1561 (92.8)	122 (7.2)	428 (86.8)	65 (13.2)	231 (81.1)	54 (18.9)
Yes	403 (78.7)	109 (21.3)	210 (77.5)	61 (22.5)	250 (62.8)	148 (37.2)
*P *value	< 0.001		0.001		< 0.001	
Cigarette smoking (%)						
No	1662 (89.7)	190 (10.3)	556 (84.5)	102 (15.5)	414 (70.4)	174 (29.6)
Yes	302 (88.0)	41 (12.0)	82 (77.4)	24 (22.6)	67 (70.5)	28 (29.5)
*P *value	0.35		0.06		0.98	
Physical Activity Score (METS/ daily) (%)						
24–36.5	434 (85.6)	73 (14.4)	136 (75.6)	44 (24.4)	159 (64.9)	86 (35.1)
36.6–44.9	1179 (89.9)	133 (10.1)	392 (85.4)	67 (14.6)	271 (73.6)	97 (26.4)
≥ 45	326 (93.1)	24 (6.9)	97 (88.2)	13 (11.8)	46 (75.4)	15 (24.6)
*P *value	0.001		0.004		0.04	
Education Year (%)						
< 6	1206 (87.3)	176 (12.7)	442 (82.6)	93 (17.4)	362 (66.5)	182 (33.5)
6-12	545 (92.1)	47 (7.9)	144 (86.7)	22 (17.5)	94 (83.9)	18 (16.1)
> 12	213 (96.4)	8 (3.6)	52 (82.5)	11 (8.7)	25 (92.6)	2 (17.4)
*P *value	< 0.001		0.45		< 0.001	
Occupation (%)						
No	997 (87.3)	145 (12.7)	322 (81.1)	75 (18.9)	305 (67.0)	150 (33.0)
Yes	967 (91.8)	86 (8.2)	316 (86.1)	51 (13.9)	176 (77.2)	52 (22.8)
*P *value	0.001		0.06		0.006	
Marital status (%)						
Single	54 (94.7)	3 (5.3)	13 (72.2)	5 (27.8)	7 (70.0)	3 (30.0)
Married	1780 (89.8)	203 (10.2)	574 (84.5)	105 (15.5)	422 (70.1)	180 (29.9)
Widowed + Divorce	130 (83.9)	25 (16.1)	51 (76.1)	16 (23.9)	52 (73.2)	19 (26.8)
*P *value	0.03		0.08		0.86	
Wealth score index (%)						
Poorest	373 (89.7)	43 (10.3)	124 (80.0)	31 (20.0)	94 (63.5)	54 (36.5)
The 2nd poorest	400 (87.1)	59 (12.9)	125 (85.0)	22 (15.0)	106 (67.9)	50 (32.1)
Middle	373 (89.9)	42 (10.1)	128 (85.9)	21 (14.1)	94 (72.3)	36 (27.7)
The 2nd richest	410 (89.7)	47 (10.3)	133 (84.2)	25 (15.8)	101 (71.6)	40 (28.4)
Richest	408 (91.1)	40 (8.9)	128 (82.6)	27 (17.4)	86 (79.6)	22 (20.4)
*P *value	0.41		0.66		0.07	
ACEI using (%)						
No	1938 (89.7)	223 (10.3)	629 (84.1)	119 (15.9)	452 (71.7)	178 (28.3)
Yes	26 (76.5)	8 (23.5)	9 (56.2)	7 (43.8)	29 (54.7)	24 (45.3)
*P *value	0.01		0.003		0.009	
ARB using (%)						
No	1909 (90.5)	201 (9.5)	599 (83.9)	115 (16.1)	409 (73.0)	151 (27.0)
Yes	55 (64.7)	30 (35.3)	39 (78.0)	11 (22.0)	72 (58.5)	51 (41.5)
*P *value	< 0.001		0.28		0.001	

CKD, chronic kidney disease; GFR, glomerular filtration rate; MDRD, Modification of Diet in Renal Disease study; METS, metabolic equivalents ACEI, angiotensin-converting enzyme inhibitor; ARB, angiotensin receptor blocker.

**Table 2:** Prevalence of CKD Based on Different Definitions

**Variables**	**Glycemic status**			***P *** **Value**
	**Normal**	**Diabetes**	**Prediabetes**	
CKD, N (%)				< 0.001
No	1964 (89.5)	481 (70.4)	638 (83.5)	
Yes	231 (10.5)	202 (29.6)	126 (16.5)	
CKD (GFR < 60 (mL/min)				< 0.001
No	2048 (93.3)	589 (86.2)	678 (88.7)	
Yes	147 (6.7)	94 (13.8)	86 (11.3)	
CKD (Alb / Cr ≥ 30 mg/g)				< 0.001
No	2098 (95.6)	538 (78.8)	718 (94.0)	
Yes	97 (4.4)	145 (21.2)	46 (6.0)	
CKD_Stage2				< 0.001
1	431 (19.6)	100 (14.6)	129 (16.9)	
2	1617 (73.6)	489 (71.6)	549 (71.9)	
3	146 (6.7)	91 (13.3)	85 (11.1)	
4	1 (0.1)	2 (0.3)	1 (0.1)	
5	0 (0.0)	1 (0.1)	0 (0.0)	
GFR (MDRD)	79.20 ± 13.34	74.99 ± 15.0	77.13 ± 14.29	< 0.001
(Mean ± SD)	6.77 ± 0.71	46.85 ± 5.69	9.32 ± 1.39	< 0.001

CKD, Chronic kidney disease; GFR, Glomerular filtration rate; MDRD, Modification of Diet in Renal Disease study.

Table 1 indicates that the prevalence of CKD was higher in men than women among individuals with normal glucose levels and those with prediabetes. However, among diabetic individuals, the prevalence of CKD was higher in women than men. CKD prevalence was higher among cigarette smokers (17.1%) than their counterparts (15.04%). Furthermore, those with an education period of less than 6 years, widowed and divorced people, unemployed people, those with low physical activity, and those belonging to the poorest WSI category were more likely to have CKD than other groups. The risk of developing CKD was greater in individuals with high blood pressure than those without it.

Table 3 shows the crude and adjusted odds ratios for various related risk factors, as determined by multivariable logistic regression. The results showed that age (OR = 1.02; 95% CI: 1.01, 1.03), male sex (OR = 6.64; 95% CI: 5.47, 8.06), dysglycemia (OR _Prediabetes_ = 1.26; 95% CI: 1.04, 1.54), ( OR _Diabetes_ = 1.77; 95% CI: 1.44, 2.18), hypertension (OR = 1.89; 95% CI: 1.48, 2.18), ARB use (OR = 1.48; 95% CI: 1.06, 2.07) and abnormal waist circumference (OR = 1.007; 95% CI: 1.00, 1.01) as independent risk factors lead to an increase in CKD.

**Table 3:** Logistic Regression Analysis of Chronic Kidney Disease and Glycemic Status in Bandare-Kong Cohort Study (n = 3642)

**Variable**	**Crude**				**Adjusted**			
	**OR**	**95% CI**		***P *** **Value**	**OR**	**95% CI**		***P *** **Value**
		**Lower**	**Upper**			**Lower**	**Upper**	
Age (y)	1.02	1.01	1.03	< 0.001	1.02	1.01	1.03	< 0.001
Gender								
Female	1	—	—	—	1	—	—	—
Male	5.50	4.73	6.40	< 0.001	6.64	5.47	8.06	< 0.001
Glycemic status								
Normal	1	—	—	—	1	—	—	—
Prediabetes	1.44	1.21	1.71	< 0.001	1.26	1.04	1.54	0.01
Diabetes	1.79	1.50	2.14	< 0.001	1.77	1.44	2.18	< 0.001
Hypertension								
No	1	—	—	—	1	—	—	—
Yes	1.93	1.67	2.23	< 0.001	1.89	1.48	2.18	< 0.001
Cigarette Smoking								
No	1	—	—	—	1	—	—	—
Yes	2.94	2.44	3.53	< 0.001	0.86	0.69	1.06	0.16
ACEI use								
No	1	—	—	—	1	—	—	—
Yes	1.96	1.34	2.87	0.001	1.39	0.89	2.18	0.14
ARB use								
No	1	—	—	—	1	—	—	—
Yes	1.99	1.56	2.56	< 0.001	1.48	1.06	2.07	0.02
Daily energy intake	0.99	0.97	1.00	< 0.001	0.99	0.97	1.00	0.39
Waist Circumference	0.11	0.99	1.00	0.11	1.007	1.00	1.01	0.04
Salt (gram/daily)	0.96	0.93	1.00	0.07	0.99	0.95	1.03	0.71

CKD, Chronic kidney disease; ACEI, Angiotensin-converting enzyme inhibitor; ARB, Angiotensin receptor blocker.

## Discussion

 The present study was performed on the first phase of the Bandare-Kong Persian cohort. It aimed to assess the epidemiological characteristics of CKD within diabetic, prediabetic, and healthy populations and to identify prognostic factors for CKD utilizing the MDRD GFR equation. The prevalence of CKD was found to be 15.3%, with 29.6% identified in diabetic individuals and 16.5% in prediabetic patients, encompassing a total of 3642 adults in Bandare-Kong. Overall, increased age, dysglycemia (diabetes or prediabetes), hypertension, and the use of ARBs were significantly associated with elevated risk of CKD in adults. These results align with previous studies offer valuable insight for shaping preventive strategies for CKD.^33^

## Prevalence

 The estimate of CKD prevalence in Asia ranges between 7.0% and 34.3%.^34^ This aligns closely with the findings of a recent systematic review and meta-analysis involving 100 studies and 6 908 440 patients, which reported a comparable prevalence of 13.4%.^35^ Notably, our CKD prevalence is significantly lower than that reported in a study conducted in Yazd, Iran, where 27.5% of 9781 participants aged 30–73 was found to have CKD.^36^

 In 2019, a study conducted in Shiraz, Iran, reported a CKD prevalence of 16.6% based on the estimated glomerular filtration rate (eGFR).^37^ In comparison, the Golestan cohort study, which is the largest cohort study in both Iran and the Middle East, estimated CKD prevalence at 23.7% in 2017. This study, led by Sepanlou et al, found a prevalence rate of 26.6% in women and 20.6% in men.^38^

 Other studies conducted in Iran show different prevalence rates for CKD. In a cross-sectional study spanning 2002 to 2005, Safarinejad et al identified a CKD prevalence of 12.6% among 17,000 adults aged 14 and older.^39^ In contrast, Najafi et al found a lower CKD prevalence of 4.6% among adults in Golestan, based on GFR measurements.^40^

 The cumulative data suggest a notable growth in CKD prevalence in Iran over the past few years, indicating a significant health concern.

###  Risk Factors

 This study observed an average age of 50.67 ± 9.84 years among individuals with CKD, revealing a higher CKD risk associated with aging. Multivariate analysis indicated a 5% increase in CKD chance for every 10-year increment in age (OR = 1.05; 95% CI: 1.04, 1.06), consistent with prior research emphasizing the age-related rise in CKD odds.^36,38,41,42^

 Comparing genders using the MDRD equation did not reveal a significant difference in CKD development (OR = 1.05, 95% CI = 0.88‒1.26). However, contrasting results from the Japanese Society for Dialysis Therapy suggested a higher likelihood of CKD in males, differing from UK and Sweden studies.^43,44^

 This study demonstrated that diabetic patients were 1.94 times more likely to develop CKD (OR = 1.94, 95% CI = 1.53‒2.46), aligning with similar findings in the literature.^45-49^

 Diabetes mellitus stands as the primary cause behind CKD and ESRD in both developed and developing nations.^50,51^ Data from the United States Renal Data System (USRDS) reveals that diabetic nephropathy is present in half of the newly diagnosed ESRD patients in the United States.^52^ In our study, the prevalence of CKD within the diabetic population reached 29.6%, surpassing the rates reported by Kumela Goro et al^53^ (26%) and a study in the United Kingdom (27.5%).^43^ Similarly, Spain documented a CKD prevalence of 31.22% among diabetic and hypertensive patients.^54^ The United States recorded a CKD prevalence of 38.3% among T2DM patients from 2007 to 2012.^55^ Discrepancies in these figures may stem from variations in study populations. Notably, low- and middle-income countries exhibit considerable CKD heterogeneity between urban and rural areas. Furthermore, the etiology of CKD in type 2 diabetic patients in low-income countries is complex, influenced by the burden of both non-communicable and communicable diseases, setting them apart from their counterparts in high-income countries.^56^

 Our investigation uncovered a noteworthy correlation between hypertension and CKD (OR = 1.75, 95% Cl = 1.40‒2.20). This finding aligns with previous studies,^57-60^ affirming hypertension as a pivotal risk factor for CKD development. In our study, 26.93% of the hypertensive population exhibited CKD. The positive impact of blood pressure control and antihypertensive medications on renal function in diabetic patients has been consistently emphasized in recent guidelines.^61^ Systemic hypertension exerts pressure on intraglomerular capillaries, contributing to glomerulosclerosis and subsequent renal function deterioration. Consequently, hypertensive patients face various risks of renal damage, as documented in previous research.^52^

 The utilization of ARBs appears to elevate the risk of developing CKD (OR = 1.38; 95% CI: 1.01, 1.89). Conversely, there was no statistically significant association found between ACEI use and the presence of CKD. The role of ARBs and ACEI in CKD remains a subject of uncertainty. The KDIGO guideline recommends renin-angiotensin system (RAS) inhibitors for managing CKD in diabetic patients.^62^ Additionally, a meta-analysis of 119 randomized trials involving CKD patients, whether or not they had diabetes, showed that both ACEIs and ARBs were effective in reducing the risk of kidney failure, or ESKD, as well as major cardiovascular events.^63^ Further, a recent trial found that discontinuing RAS inhibitors did not lead to significant changes in eGFR or a difference in the long-term rate of eGFR decline in patients with advanced and progressive CKD.^64^

 The current study found no evidence linking CKD with either cigarette smoking or hookah use, which contrasts with previous research.^36,65^ Earlier, a systematic review and meta-analysis of 15 cohort studies had identified cigarette smoking as an independent risk factor for developing CKD.^65^ Additionally, the research by Dehghani et al also recognized smoking as a risk factor for CKD.^36^

 Furthermore, this study did not find a statistically significant correlation between CKD and physical activity. This is in contrast to Shi et al who indicated an inverse relationship between overall physical activity and the risk of CKD.^66^

## Limitations

 Although our investigation contains valuable insight regarding the prevalence and risk factors of CKD among subjects with different glycemic statuses, it has some limitations. Firstly, our subjects were collected from a coastal area in southern Iran. So, it may not completely represent the entire country, which could limit the generalizability of the study findings. Secondly, single urine creatinine and albumin assay was used for CKD classification with no follow-up test conducted after three months. This could decrease the accuracy of our CKD classification. A more precise monitoring protocol would increase accuracy. Thirdly, although our data was adjusted for different known confounders, some unmeasured variables such as physical activity, environmental exposures, dietary habits, and genetic predispositions might affect the association between glycemic status and CKD. Future studies should consider a broader range of potential confounders for a more comprehensive understanding.

## Conclusion

 The findings underscore that the primary risk factors for CKD are potentially modifiable. By effectively managing blood pressure and blood sugar, particularly in individuals with diabetes and hypertension, there exists the potential to mitigate the occurrence of CKD. Given the age-related increase in CKD prevalence, it is advisable to incorporate CKD screening into the primary healthcare system, particularly targeting individuals aged 50 and above.

 This proactive screening approach facilitates the early detection of CKD, enabling interventions to prevent its progression to advanced stages requiring kidney replacement therapy. Therefore, strategic decisions should be implemented to improve both healthcare professionals’ and the general population’s knowledge and awareness of the importance of early diagnosis and prevention of CKD.

 This concerted effort aims not only to improve CKD prognosis but also to alleviate mortality rates and reduce the financial strain caused by kidney failure.
